# Selenium Content of the Gonads of the Domestic Dog (*Canis lupus f. familiaris*) in Relation to Sex, Age, and Reproductive Disorders

**DOI:** 10.3390/ani15233502

**Published:** 2025-12-04

**Authors:** Ewa Skibniewska, Marta Kołnierzak, Bartosz Skibniewski, Iwona Lasocka, Michał Skibniewski

**Affiliations:** 1 Department of Biology of Animal Environment, Institute of Animal Science, Warsaw University of Life Sciences, Ciszewskiego Street 8, 02-786 Warsaw, Poland; marta_kolnierzak@sggw.edu.pl (M.K.); iwona_lasocka@sggw.edu.pl (I.L.); 2 One Health Section, The Scientific Society of Veterinary Medicine Students, Faculty of Veterinary Medicine, Warsaw University of Life Sciences, Nowoursynowska Street 159, 02-776 Warsaw, Poland; s206916@sggw.edu.pl; 3 Department of Morphological Sciences, Institute of Veterinary Medicine, Warsaw University of Life Sciences, Nowoursynowska Street 159, 02-776 Warsaw, Poland

**Keywords:** selenium, dogs, testicles, ovaries, age, health status

## Abstract

**Simple Summary:**

Selenium, as a component of selenoproteins, affects the activity of the immune system and the detoxification of heavy metals from the body, thereby preventing disturbances to homeostasis and the accumulation of toxic metals. This study aimed to assess the selenium content in the gonads of dogs from the Warsaw metropolitan area, taking into account gender, age, and health status in terms of homeostasis disorders leading to pathologies in the reproductive organs of the individuals examined. Higher selenium levels were observed in the gonads of male dogs. The lowest selenium content was found in both males and females in the oldest age group. In dogs with homeostasis disorders, the lowest selenium levels were observed in the ovaries of females with pyometra and in males with atrophic testicles.

**Abstract:**

Appropriate selenium (Se) levels contribute to the weakening of the effects of cellular peroxidation and have a protective function, ensuring the effectiveness of the defense against various diseases. The study aimed to determine the selenium content in the gonads of dogs from the Warsaw metropolitan area, taking into account the influence of gender, health status, and individual. The research material consisted of testicular and ovarian samples obtained from 86 animals during routine surgeries. Selenium content in the samples was determined using fluorometric spectrometry. The mean selenium content in the gonads of all components was found to be 0.43 mg·kg^−1^ wet weight. In males, it was 0.49 mg·kg^−1^ wet weight, while in females, it was 0.39 mg·kg^−1^ wet weight. The lowest selenium concentration was found in animals of both sexes in the group of individuals older than 7 years. In individuals with disturbed selenium homeostasis, 0.34 mg·kg^−1^ wet weight occurred in the ovaries of females with pyometra, and in males, 0.41 mg·kg^−1^ wet weight occurred in individuals with testicular atrophy.

## 1. Introduction

Selenium (Se) was recognized as an essential nutrient for the body in 1957 [1]. Since that discovery, knowledge about selenium in humans and various animal species has been gradually supplemented. It is already known that selenium is a microelement that can be found in every mammal’s tissues, and it acts as an antioxidant and plays a role in the synthesis of DNA, thyroid metabolism, and reproduction [2,3,4,5]. Its presence is particularly important to ensure the normal function of the male and female reproductive systems, for the development of gametes, fertilization, and maintenance of pregnancy [3].

Selenium, as a component of selenoproteins, has an effect on the activity of the immune system and detoxicity of heavy metals from the body, which prevents homeostasis disturbances and the accumulation of Cd and Hg [6,7,8,9,10]. The biological role of selenium at the cellular level is primarily related to its presence in the active center of glutathione peroxidase (GPX), one of the enzymes involved in the body’s defense against oxidative stress [11,12]. Glutathione peroxidase (GPX) in the testes is involved in sperm DNA condensation and is a component of mature spermatozoa [13,14]. Like SePP (the plasma protein that delivers selenium to the testes), GPX plays a key role in male fertility, maintaining adequate viability and motility of sperm [13,14,15,16,17]. In females, selenium supplements have been shown to improve fertility and reduce the incidence of endometrial lesions and ovarian cysts [18]. Domosławska et al. [19] found that supplementation with selenium and vitamin E has a positive effect on semen quality in dogs with lowered semen quality. Panahi et al. [20] showed that the addition of selenium nanoparticles to dog semen at concentrations of 0.1 and 0.5 μg can improve its parameters after storage in liquid media. For that reason, selenium could become a factor routinely considered when dealing with infertile and subfertile dogs [5]. In conditions of vitamin E and selenium deficiency, a negative impact on processes involved in female reproductive function, such as ovulation rate, uterine motility, sperm motility and transport, conception rate, and embryo viability, has also been shown [21].

Poland is a selenium-deficient area, and selenium supplements are widely used in fertilizers, livestock feed, and supplements for humans and companion animals [3,22,23,24]. Clinical signs of Se deficiency in dogs include muscular weakness, subcutaneous edema, anorexia, depression, dyspnea, depressed response to mitogenic stimulation, and it also seems to affect male fertility [5,25]. Pereira et al. [4] indicated that dietary supplementation with selenium (Se) is a common practice to ensure dogs’ requirements, but the Se source does matter, and organic Se forms indicate better retention. The element is absorbed from the diet in the form of organic compounds such as selenites (Me_2_SeO_3_) and selenates (Me_2_SeO_4_) or organic combinations with amino acids in the form of selenomethionine (SeMet) and selenocysteine (SeCys). Organic selenium is mainly obtained from meat. Inorganic selenium, mainly in the form of sodium selenate, is supplied in supplementation form with commercial dog food [5,26]. Van Zelst et al. [2] noted that the type of dog food (canned or kibble) must also be considered as they differ in terms of the biological activity of Se. In dogs, selenium is primarily excreted in the urine and can be measured by the selenium-to-creatinine ratio [5,27]. The highest concentrations of selenium are found in the testicles, renal cortex, and pituitary gland, followed by the thyroid, adrenal glands, ovaries, liver, spleen, and cerebral cortex [14,28]. Particularly important is its deposit in the male gonads, which increases with sexual maturation. In juveniles, it accounts for about 5% of the total content in muscle and liver. During sexual maturity, its level increases to 10% [29]. Knowledge of canine infertility is still insufficient. The etiology of diseases leading to poor semen quality often remains unknown, and disorders of this nature can occur at any age. Spermatogenesis relies on numerous internal and external factors that influence testicular function. The relationship between oxidative stress, antioxidants, and fertility has been confirmed in numerous studies in both humans and animals [12,30,31].

Selenium deficiencies are primarily caused by an insufficient selenium supply. However, they may arise as a result of disturbances in their transport and inadequate synthesis of selenoproteins, due to disturbed homeostasis [10,29]. The initial symptoms of dietary selenium deficiency are nonspecific, including recurrent infections, joint pain, pale skin, accelerated aging, and brittle nails and hair. In selenium deficiency states, the body first supplies the necessary amount of selenium to the testes, then other organs are supplied [29]. Selenoprotein (SePP) is then inactivated, resulting in infertility in males due to structural defects in sperm and loss of sperm motility. Selenium levels in the testes are retained until all the selenium stored in the tissues is depleted [10,15,32,33].

There are few reports in the available literature on the content of micro- and macronutrients in the tissues of the canine reproductive system; the present study will expand the state of knowledge in this area.

The domestic dog (*Canis lupus f. familiaris*) appears to be an appropriate research model for studying human-related issues. As a companion animal, it shares the same habitat with humans and is therefore exposed to the same detrimental factors that contribute to many diseases in both species, such as mammary tumors, prostate cancer, cardiomyopathy, epilepsy, and hip dysplasia [3,33,34]. The aim of this study was to determine the selenium content in the gonads of domestic dogs from the Warsaw metropolitan area and to determine the influence of age, sex, and selected reproductive organ pathologies on its concentration in the testes and ovaries.

## 2. Materials and Methods

The study material consisted of testes and ovaries of pet dogs obtained during scheduled gonadectomy procedures performed at selected veterinary clinics in Warsaw between 2017 and 2019. Material was collected from a total of 86 individuals of both sexes (males n = 38, females n = 48). Healthy individuals (males, n = 16; females, n = 27) and those with disturbed homeostasis (males, n = 22; females, n = 21) were selected for the study. In males with identified homeostasis disorders, cryptorchid (c) individuals (n = 11), and dogs with testicular atrophy (a) (males n = 11) were selected. For females with impaired homeostasis, there were individuals from ovarian cystic lesions (cl) (n = 11) and females suffering from pyometra (p) (n = 10). Both in the group of healthy animals and in the group of individuals with impaired homeostasis, the dogs were divided into three age groups: young dogs (up to 1.5 years of age), mature individuals (1.5 to 7 years), and geriatric individuals (above 7 years of age).

The ovaries and testes were harvested whole and packed into polyethylene containers, which were then refrigerated and transported to the laboratory for further processing. Until assays were performed, the tissues were kept in a deep-frozen state in polythene bags at −20 °C. After thawing, the organs were prepared to collect adequately representative samples. Testicular parenchyma and ovarian sections, weighing approximately 0.5–1.0 g of wet tissue, were collected for analysis, as described by Skibniewska and Skibniewski [31]. All samples were analyzed three times, and the results obtained were the arithmetic means of the three analyses. The elemental content was expressed in milligrams per kilogram (mg∙kg^−1^) wet weight.

Based on information obtained from the 3rd Local Ethical Committee in Warsaw, according to current legislation, research using animal tissues collected during routine prophylactic procedures and procedures performed for medical indications, treated as waste tissue, does not require additional authorization. Selenium content was determined by spectrofluorimetry using a Shimadzu RF-5001 PC spectrophotometer (detection limit 0.003 µg·g^−1^). Mineralization of the samples was performed according to the method described by Pilarczyk et al. [34]. Balances were placed in 100 cm^3^ spherical flat-bottomed ground flasks to which 3.5 cm^3^ of concentrated V-valent nitric acid (65% from Chempur) was added.

Samples were allowed to stand for 24 h and then mineralized at 230 °C for 3 h. After cooling, 3 cm^3^ of perchloric acid (VII) (70% from Chempur) was added and mineralized at 310 °C for 20 min. The mineralized samples (including the reagent sample) were hydrolyzed using 3 cm^3^ of 9% hydrochloric acid (HCl) and heated in a water bath for 20 min. The samples were then rapidly cooled to room temperature. To the hydrolyzed samples, 10 cm^3^ of EDTA versenoic acid, 2 cm^3^ of ammonium hydroxide NH_4_OH (1:1), and a few drops of cresyl red were added, after which the pH was set between 1.4 and 1.8. Further analyses were performed under artificial yellow light to avoid degradation of the versenoic acid. 5 cm^3^ of DAN (2,3-diaminonaphthalene) was added to the samples. The flasks were closed with stoppers and placed in a hothouse heated to 50 °C for 30 min. Then 6 cm^3^ of cyclohexane was added to the samples, after which the samples were shaken on a shaker at 500–600 rpm for 15 min. Selenium concentration was measured in the resulting cyclohexane layer (at 518 nm, the excitation wavelength was 376 nm). The selenium content was determined using a SHIMADZU RF-5001 PC spectrophotometer.

The correctness of the analytical procedure was checked by determining the selenium concentration in the reference material NCS ZC 71001 (Beef Liver) (China National Analysis Center for Iron and Steel, Beijing, China). In parallel with each run (30 samples), samples of the reference material (n = 3) and reagent samples (blank) (n = 4) were analyzed chemically. The selenium concentration of the certified reference material is shown in Table 1.

Statistical analysis of the results, including correlations across groups, was performed using Statistica 13.3 (TIBCO Inc. ™ StatSoft, Krakow, Poland). The normality of the distribution of the variables was tested using the Shapiro–Wilk W test. The data did not have a normal distribution, so the Mann–Whitney U test was used to compare differences between groups at a significance level of *p* ≤ 0.05 and *p* ≤ 0.01. Spearman’s r correlation coefficients were calculated at a significance level of *p* ≤ 0.05 and *p* ≤ 0.01 to determine intergroup correlations.

## 3. Results

Based on the results obtained, the selenium content in the gonads of the dogs studied ranged from 0.304 to 0.74 mg∙kg^−1^ wet weight of tissue. Detailed data are presented in Table 2. The mean selenium content in the testes was higher compared to its content found in the ovaries. Both values were statistically significantly different.

### 3.1. The Impact of Age on Se Concentration in the Gonads of Dogs

In males, the highest selenium content was recorded in the group of animals aged 1.5 to 7 years, while the lowest was recorded in dogs in the geriatric group, older than 7 years (Table 3). Lower values were recorded in females across each age group. Similarly to males, the lowest gonadal selenium contents were recorded in the geriatric group of females. The highest average content of this element was recorded in the gonads of females up to 1.5 years of age, classified in the first age group (Table 4).

**Figure 1:**
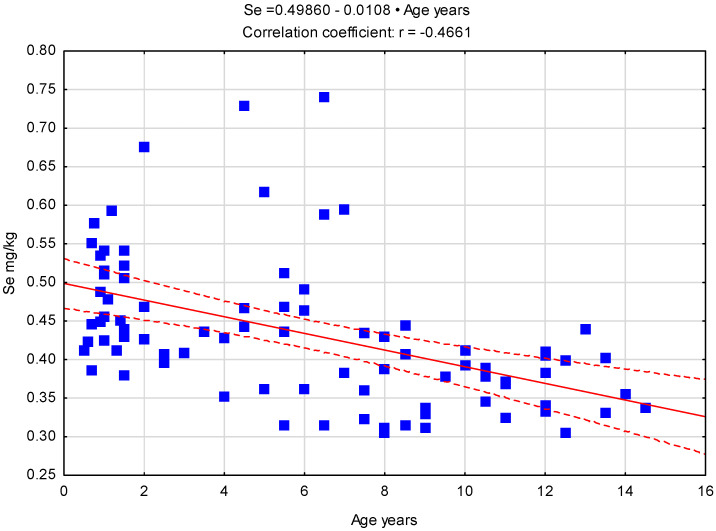
Correlation between age and Se content in all dogs tested, n = 86. Blue squares—values for certain individuals of both sexes, red line—regression line, red dash lines 0.95 confidence interval.

An analysis of the correlation between age and Se content in the gonads of individuals of both sexes allows us to conclude that its content decreases as the animals age (Figure 1).

**Figure 2:**
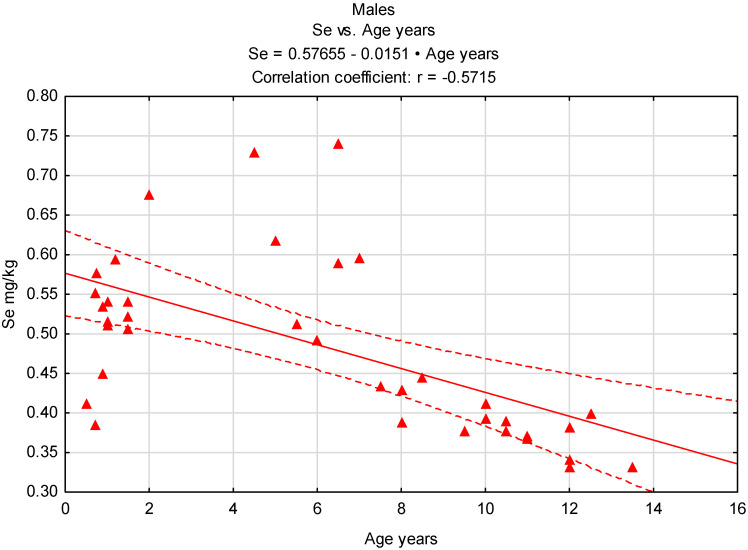
Correlation between age and Se content in males, n = 38. Red triangles—values for males tested, red line—regression line, red dash lines 0.95 confidence interval.

**Figure 3:**
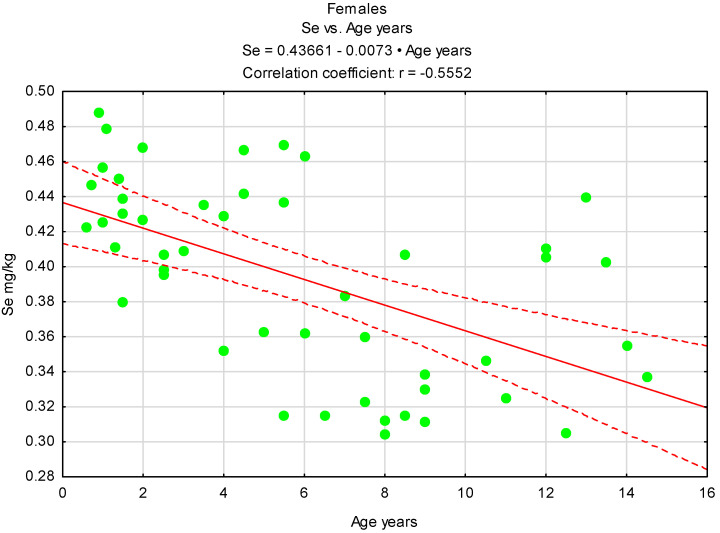
Correlation between age and Se content in females, n = 48. Green circles—values for females tested, red line—regression line, red dash lines 0.95 confidence interval.

It should be noted that in the case of males, the cut-off age after which there is a clearly visible decrease in Se content in the testes is their seventh year of life, despite the fact that these values are higher than those recorded in the gonads of females (Figure 2 and Figure 3).

### 3.2. The Effect of Pathological Lesions on Se Gonadal Concentration

The relationship of pathological changes in the gonads to selenium content in male specimens is shown in Table 5, while the values for females are presented in Table 6. It is worth noting that the incidence of pathological changes is also related to an individual’s age. In the group of youngest males, only 2 individuals were clinically healthy, while the remaining 11 individuals were cryptorchids that underwent routine gonadectomy to remove undescended testes. The second pathology analyzed was testicular atrophic changes secondary to other disease processes. In the second age group, only 1 individual showed features of testicular atrophy, while in the third age group, there were as many as 10 individuals. The highest mean Se content was found in clinically healthy individuals. The second group consisted of cryptorchid specimens, in which the Se content was only slightly lower than in healthy specimens. In contrast, animals with atrophic testicular lesions had a low Se content, which was statistically significantly different from that of the healthy males (Table 5).

Among the females, there were no statistically significant differences between the study groups. The Se content of the ovaries was more homogeneous despite the different pathological changes, so it can be concluded that both ovarian follicle cysts and pyometra are not significantly associated with changes in Se levels in ovarian tissue.

## 4. Discussion

Biogenic elements, such as selenium, which are considered essential, play a crucial role in maintaining homeostasis. Its presence in the body determines, among other things, the correct course of metabolic processes in the gonads, both in terms of germ cell production and in relation to their endocrine function [35,36,37,38,39,40]. The selenium content in the body depends on various factors and is subject to dynamic changes. Establishing relationships in terms of elemental balance appears to be crucial for explaining systemic disorders and therapeutic solutions undertaken. Selenium status in the ovaries and testes not only reflects the overall availability of the element in the body but also has a significant impact on fertility, as it supports reproductive function. In the literature, studies on the association between biogenic metals and gonadal function, as well as concomitant pathologies, in human companion carnivores are scarce [31,38]. The effect of selenium is dependent on the content of other metals in the tissues. Elemental deficiency or excess can lead to oxidative stress and disorders of homeostasis. All dietary supplements should therefore be used with caution, as they may cause harmful side effects. In dogs, improvements in semen parameters have been observed following administration of various combinations of vitamin E, selenium, zinc, and n-3 polyunsaturated fatty acids (PUFAs) [12]. Domoslawska et al. [30] showed that antioxidants act synergistically when given together. Selenium supplementation, together with vitamin E, resulted in a rapid increase in sperm motility in dogs after only 30 days of treatment. The improvement in sperm concentration in the ejaculate lasted for 60 days, which was related to the duration of spermatogenesis in dogs. In clinical practice, selenium has been demonstrated to play a crucial role in reproduction. Therefore, routine testing of plasma selenium levels should be considered when diagnosing causes of canine infertility [5]. Żarczyńska et al. [6] report that selenium deficiency contributes to, among other things, ovarian cyst formation and increased embryo mortality during the first 3–4 weeks after insemination. Selenium deficiency is also thought to induce changes in the LH receptors of Leydig cells, which affects testosterone secretion. In selenium-deficient rodents, oligospermia, a decrease in sperm motility, and an increase in the number of abnormal spermatozoa were observed. No reproductive abnormalities were observed in these individuals; however, their offspring exhibited slower growth, a lack of body hair, and muscle weakness. In pregnant females, low selenium concentrations did not affect the final length or body weight of the newborns [29].

Although this element is essential, its excess has a toxic effect. Chronic Se toxicity (selenosis) in dogs is indicated by impaired growth and restricted food intake, anorexia and stunted growth, nausea and vomiting, diarrhea, apprehension, respiratory stimulation, and cardiovascular changes, but there are no naturally occurring cases known [5,25,41]. Neurological disorders may also be associated with Se imbalance. Rosendahl et al. [42] revealed higher Se concentration in whole blood and hair of dogs diagnosed with idiopathic epilepsy compared to healthy dogs. Selenium deficiency affects all animal species; however, the accurate assessment of selenium status in dogs remains poorly understood. Van Zelst et al. [27] showed that urinary Se to creatinine ratio, serum Se, and serum and whole blood glutathione peroxidase can be used as biomarkers of selenium status in dogs, but the first two mentioned responded faster to decreased dietary Se than the other parameters (mRNA expression and serum copper, creatine kinase, triiodothyronine: thyroxine ratio and hair growth).

The existing narrow margin between necessary and toxic dose forces supplementation to be carried out with extreme caution [12,18]. The maximal legal limit for total Se content in dog food is 0.5 mg∙kg^−1^ DM [43]. The minimal limit for early growth and reproduction, as well as for late growth, is 0.40 mg∙kg^−1^ DM [5].

There are reports in the available literature on the role of selenium in regulating testicular and ovarian function; however, these are limited to an analysis of its effects on reproductive processes in humans, livestock, and laboratory animals. Several publications have been written on companion animals [31,38,44,45,46,47,48].

The selenium values in the gonads of females (Table 4) obtained in our study are similar to the value of 0.38 mg∙kg^−1^ wet weight recorded by Forte et al. [48] in the ovaries of females living in small towns, and lower compared to the value of 0.42 mg∙kg^−1^ recorded by these authors in the ovaries of dogs from large cities.

Unlike herbivorous livestock (horses, cattle, sheep, or goats) or omnivorous species, dogs, as carnivores, maintain higher selenium levels, ranging from 1.90 to 5.1 µmol∙L^−1^ in serum/plasma and from 3.1 to 12.7 µmol∙L^−1^ in whole blood [5]. Pilarczyk et al. [45] recorded serum selenium concentrations in dogs ranging from 0.169 to 0.273 mg∙mL^−1^. The results obtained in their own study indicate that selenium content in the gonads of dogs was lowest in the group of the oldest individuals. A similar relationship between the decrease in selenium and animal age was found by Skibniewska and Skibniewski [31] when analyzing the content of this element in the gonads of domestic cats. It is likely that the lower selenium values in the body of older individuals are linked to, among other things, to a weakening of the immune system function and of the mechanisms protecting cellular structures from the effects of peroxidation, which can also be linked to their higher incidence of various diseases, e.g., diabetes, circulatory disorders, mammary gland cancer, prostate cancer [29,47,48]. Selenium has been shown to play an important role in cancer prevention. In experiments conducted on cell cultures, selenium has been shown to inhibit the growth of cancer cells and induce apoptosis of various cancer cell lines isolated from dogs [5]. Older, uncastrated dogs often suffer from disorders related to prostate function; hence, it is recommended to feed them Se-enriched food to reduce their risk of prostate cancer by up to 63% [45,49]. Alexander and Olsen [50] also report that in humans, high doses of selenium reduce the risk of breast, lung, oesophageal, stomach, and prostate cancer. However, these authors did not show such a relationship with colorectal cancer, bladder cancer, and skin cancer.

In our study, we found that the selenium content in the testes of dogs with impaired homeostasis was lower compared to the value found in the gonads of healthy males. The highest mean content of this element. was recorded in healthy individuals, followed by cryptorchids, in which the Se content was not significantly different from that of healthy animals. It should be noted, however, that the cryptorchids represented young animals in the first age group. Studies in males of other mammalian species indicate that the Se content of the testes increases at sexual maturity. Analyzing the pharmacokinetics of Se-containing compounds, it was found that there is a significant difference between the levels of selenium in the male and female gonads, as well as a difference in the distribution time of this element, which is taken up by the ovaries quickly, but does not reach the level recorded in the testicular tissue. In the case of the ovaries, the level of absorbed Se decreases steadily over time, while its uptake by the testes occurs with a delay and consequently reaches a much higher content than recorded in the female gonads [51]. Deposited in the testes, selenium protects the sperm mitochondria from oxidative processes. Selenium, in the form of selenocysteine, is the catalytic center of the antioxidant glutathione peroxidase; hence, there is a higher concentration of selenium in the gonads in males than in females [52].

Our own results indicate that the mechanism of selenium deposition in the male gonads is similar in normally positioned testes and in testes that have not descended. The lowest Se content in atrophic testes is probably due to metabolic disturbances within the organ, as is the case with other pathologies. It may be due to a reduction in its systemic content. Zentrichová et al. [5] observed that dogs diagnosed with malignant tumors have significantly lower serum selenium concentrations than healthy individuals and dogs with other pathologies. In contrast, Enginler et al. [53] observed no significant differences in serum selenium concentrations between dogs with mammary tumors and healthy individuals. For females with impaired homeostasis, the authors of the study also did not register this relationship. It can be observed that the selenium content in the ovaries with cystic lesions was slightly higher compared to the value recorded in the gonads of healthy females. Nevertheless, it is worth noting that the two groups did not differ significantly statistically. The slight differences in Se content observed may be the result of various factors, including diet, age, breed, and current hormonal status of the individuals. The authors of the study acknowledge certain limitations resulting from the study, which included a large number of animals that were patients at veterinary clinics; therefore, many variables, such as feeding patterns, were not taken into account. The study is population-based and aims to determine the Se content in a diverse group of individuals, varying in terms of sex, age, and health status. Studies available in the literature suggest that companion animals (dogs, cats) are a suitable indicator model for humans by sharing their habitat. However, it is worth noting that there are some limitations. The content of elements in tissues is subject to dynamic changes resulting from the action of various factors on the body, including age, sex, health status, and diet [31,38,48,54].

## 5. Conclusions

Monitoring the health of dogs in the context of reproductive system pathologies, including the analysis of selected trace elements, is important because reproductive system diseases often occur in unneutered mature individuals, posing a serious clinical problem. Based on the study, it can be concluded that the Se content in the gonads of males is significantly higher than in the ovaries. The age of the individual appears to be a significant determinant of Se deposition in the testes, and this is consistent in both healthy and cryptorchid individuals. It was found that a marked decrease in Se content in males occurs at 7 years of age. In the case of females, a similar phenomenon is observed, characterized by a clear decrease in Se content with age. Pathological changes in the genital organs, such as ovarian cystic lesions or pyometra, appear to be unrelated to the Se content of the ovaries.

## Figures and Tables

**Table 1:** Selenium concentration in the reference material.

Certified Concentration	Detected Concentration	Recovery Percentage
0.56 µg∙g^−1^	0.511 µg∙g^−1^	91%

**Table 2:** Selenium content in the gonads of dogs according to the sex of animals tested (mg∙kg^−1^ w.w.).

	N	Average ± SD	Median	Min.	Max.	Q25	Q75
All animals	86	0.434 ± 0.09	0.412	0.304	0.739	0.368	0.469
Males	38	0.486 ^A^ ± 0.11	0.470	0.331	0.739	0.387	0.551
Females	48	0.394 ^B^ ± 0.05	0.405	0.304	0.488	0.342	0.438

N—number of individuals; SD—standard deviations; Min.—minimum; Max.—maximum; Q25—lower quartile; Q75—upper quartile; A, B—statistically significant differences at *p* ≤ 0.01.

**Table 3:** Se content in the gonads of males representing different age groups (mg∙kg^−1^ w.w.).

Age Group	N	Mean ± SD	Median	Min.	Max.	Q25	Q75
Below 1.5 years old	13	0.511 ^A^ ± 0.060	0.521	0.386	0.593	0.535	0.576
1.5–7 years old	9	0.631 ^B^ ± 0.093	0.617	0.492	0.740	0.553	0.646
Above 7 years old	16	0.385 ^C^ ± 0.033	0.385	0.331	0.444	0.377	0.389

N—number of individuals; SD—standard deviations; Min.—minimum; Max.—maximum; Q25—lower quartile; Q75—upper quartile; A, B, C—statistically significant differences at *p* ≤ 0.01.

**Table 4:** Se content in the gonads of females representing different age groups (mg∙kg^−1^ w.w.).

Age Group	N	Mean ± SD	Median	Min.	Max.	Q25	Q75
Below 1.5 years old	11	0.439 ^A^ ± 0.030	0.439	0.380	0.488	0.422	0.456
1.5–7 years old	19	0.407 ^AB^ ± 0.049	0.409	0.315	0.469	0.362	0.442
Above 7 years old	18	0.351 ^B^ ± 0.042	0.338	0.304	0.440	0.315	0.402

N—number of individuals; SD—standard deviations; Min.—minimum; Max.—maximum; Q25—lower quartile; Q75—upper quartile; A, B—statistically significant differences at *p* ≤ 0.01.

**Table 5:** Se content in the gonads of males with different health state (mg∙kg^−1^ w.w.).

Health State	N	Mean ± SD	Median	Min.	Max.	Q25	Q75
Healthy	16	0.532 ^A^ ± 0.139	0.523	0.341	0.740	0.388	0.646
Cryptorchid	11	0.503 ^AB^ ± 0.062	0.516	0.386	0.594	0.450	0.541
Atrophic	11	0.405 ^B^ ± 0.071	0.392	0.331	0.589	0.368	0.429

N—number of individuals; SD—standard deviations; Min.—minimum; Max.—maximum; Q25—lower quartile; Q75—upper quartile; A, B—statistically significant differences at *p* ≤ 0.01.

**Table 6:** Se content in the gonads of females with different health states (mg∙kg^−1^ w.w.).

Health State	N	Mean ± SD	Median	Min.	Max.	Q25	Q75
Healthy	27	0.406 ^A^ ± 0.056	0.425	0.305	0.488	0.355	0.442
Pyometra	10	0.341 ^A^ ± 0.028	0.342	0.304	0.396	0.315	0.362
Cystic lesions	11	0.411 ^A^ ± 0.045	0.405	0.330	0.470	0.383	0.463

N—number of individuals; SD—standard deviations; Min.—minimum; Max.—maximum; Q25—lower quartile; Q75—upper quartile; A—statistically significant differences at *p* ≤ 0.01.

## Data Availability

All data generated or analyzed during the study are included in this published article. The datasets used and/or analyzed in the current study are available from the corresponding authors upon reasonable request.
